# Impact of Structural Observables From Simulations to Predict the Effect of Single-Point Mutations in MHC Class II Peptide Binders

**DOI:** 10.3389/fmolb.2021.636562

**Published:** 2021-03-30

**Authors:** Rodrigo Ochoa, Roman A. Laskowski, Janet M. Thornton, Pilar Cossio

**Affiliations:** ^1^ Biophysics of Tropical Diseases, Max Planck Tandem Group, University of Antioquia UdeA, Medellin, Colombia; ^2^ European Molecular Biology Laboratory, European Bioinformatics Institute (EMBL-EBI), Cambridge, United Kingdom; ^3^ Department of Theoretical Biophysics, Max Planck Institute of Biophysics, Frankfurt am Main, Germany

**Keywords:** MHC class II, single-point mutation, structural bioinformatics, simulations, binding

## Abstract

The prediction of peptide binders to Major Histocompatibility Complex (MHC) class II receptors is of great interest to study autoimmune diseases and for vaccine development. Most approaches predict the affinities using sequence-based models trained on experimental data and multiple alignments from known peptide substrates. However, detecting activity differences caused by single-point mutations is a challenging task. In this work, we used interactions calculated from simulations to build scoring matrices for quickly estimating binding differences by single-point mutations. We modelled a set of 837 peptides bound to an MHC class II allele, and optimized the sampling of the conformations using the Rosetta backrub method by comparing the results to molecular dynamics simulations. From the dynamic trajectories of each complex, we averaged and compared structural observables for each amino acid at each position of the 9°mer peptide core region. With this information, we generated the scoring-matrices to predict the sign of the binding differences. We then compared the performance of the best scoring-matrix to different computational methodologies that range in computational costs. Overall, the prediction of the activity differences caused by single mutated peptides was lower than 60% for all the methods. However, the developed scoring-matrix in combination with existing methods reports an increase in the performance, up to 86% with a scoring method that uses molecular dynamics.

## Introduction

The Major Histocompatibility Complex (MHC) class II is a key receptor responsible for recognizing fragments of proteins belonging to external pathogens, as well as recognizing human proteins that can boost the emergence of autoimmune events and immunological processes (Wieczorek et al., 2017). The structures of multiple MHC class II alleles have been elucidated. They are composed of α and β chains split into four sub-units, two of them forming a groove where the peptides bind (Bjorkman, 2015) (see Supplementary Figure S1). The peptides contain a core region, which is a fragment of nine amino acids responsible to stabilize the peptide-MHC class II interaction. The peptide-core binds in four key pockets of the receptor that are formed between the α and β chains (Unanue et al., 2016). The available structures of MHC class II bound to peptides provide information about the binding poses, which are commonly in a polyproline II-like extended conformation (Bermúdez et al., 2014). Understanding the preference of amino acids for certain positions is relevant to comprehending how epitopes can trigger adaptive immune responses (Unanue et al., 2016). Moreover, this structural information allows us to study the physicochemical interactions within key pockets in the binding groove, which is crucial to stabilizing the complexes (Yeturu et al., 2010).

These structural insights are usually not included in the prediction tools of peptides binding to the MHC class II. The lack of structural and dynamical representations of the complexes, as well as the demand on computational resources, are some of the limitations (Zhang et al., 2010). Instead, researchers have focused on generating profiles and motifs of sequences using information from bioactivity datasets (Wang et al., 2008). The main purpose of these tools is to rank peptide-binders by their predicted affinities, and associate the values to a potential immunological response. Among the available approaches, the most common ones are machine learning models trained with a diverse set of peptides bound to different MHC class II alleles (Andreatta et al., 2015; Peters et al., 2020). Some other researchers have focused on creating position-specific scoring matrices that can be implemented to select peptide candidates through simple bioinformatics pipelines, and to predict which core region of the peptide is responsible for the interaction with the main pockets of the receptor (Rapin et al., 2008). The available tools cover a diverse set of MHC class II alleles, providing clues for researchers working on the design of vaccines and immunotherapies (Nandy and Basak, 2016).

One particular challenge about the binding predictions is to evaluate affinity differences for single-point mutations on the peptide. Efforts have been focused to understand the impact of such mutations in the context of protein function, participation in molecular pathways and changes in their physico-chemical properties (Bogan and Thorn, 1998; Tokuriki et al., 2007; Hopf et al., 2017). From a structural perspective, coordinates can be used as input to predict the side chain conformations of the mutated amino acids, and assess their impact from a stability or binding perspective (Li et al., 2014). In the case of MHC class II, sequence-based strategies can be implemented to predict these activity differences, but structural and dynamical insights about the mechanisms behind these modifications are also relevant (Kuhlman and Bradley, 2019; Aranha et al., 2020). Many of these methods rely on energy evaluations to check differences in terms of solvent exposure, generation of hydrogen bonds, electrostatics contributions, backbone and side chain flexibility, and weak interactions such as van der Waals (Sammond et al., 2007; Slutzki et al., 2015; Barlow et al., 2018). Understanding the main drivers of these affinity differences is relevant for the design and discovery of novel peptide binders.

Methods using structural and dynamical information can be implemented to assess the role of the peptide/receptor conformations in the binding affinity and stability (Antunes et al., 2018; Lanzarotti et al., 2018). For MHC class II, the crystal structures show that peptides tend to bind in similar conformations for the available alleles (Wieczorek et al., 2016). Therefore, these structures can be used as templates to model other peptides bound to the receptor, and enable the study of how modifications can affect the binding from a physicochemical perspective (Ochoa et al., 2019). These models can be subjected to conformational sampling to analyze the fluctuations of the complexes in equilibrium (Ferrante et al., 2015) and score the most favourable conformations (Cossio et al., 2012; Sarti et al., 2013).

Among the sampling approaches, molecular dynamics (MD) has proved to be a useful way of studying the conformational space of peptides bound to MHC class II structures (Omasits et al., 2008; Ochoa et al., 2019). However, the scalability is limited by the required computational resources if large sets of peptides are analyzed. One option is to implement Monte Carlo algorithms to obtain representative structures of the complexes in equilibrium (King and Bradley, 2010). This is the case of the backrub method from Rosetta, where the backbone flexibility is modelled based on observations from high-resolution crystal structures (Smith and Kortemme, 2008). The movements are mainly backbone rotations around the axes of C_*α*_ atoms that are accepted using a Metropolis criterion based on the minimization of a bond-angle penalty imposed by the chosen force fields (Smith and Kortemme, 2010). The trajectories provide information on the system’s intrinsic flexibility, solvent accessibility and the main interactions (e.g., hydrogen bonds, non-bonded contacts) formed by the amino acids.

In this work, we evaluated how two kinds of molecular interactions can aid in the prediction of the affinity-differences for single modifications in the core region of a set of MHC class II peptide binders. For this purpose, we created a set of scoring matrices, as is typically done using sequence analysis, but here derived from structural observables from simulations of a large set of peptides/MHC class II complexes. The matrices allow the estimation of binding differences caused by single-point mutations, and complement current state-of-the-art methods to improve the predictions. We modelled a large set of peptides with binding data available for one representative MHC class II allele. Then, we sampled the conformational space using the backrub method optimized to reproduce the finite-temperature ensemble from molecular dynamics simulations. Hydrogen bonds and contact interactions were used to calculate the scoring-matrices SM-HB and SM-C, respectively, from the structural descriptors per core position in the peptide. The magnitude and stability of these observables were associated to binding differences of single-modified peptides.

In addition, five other approaches, having a wide range of computational costs, as well as accuracy, were assessed to predict the binding differences. Specifically, two sequence-based methods were implemented, which involve the use of a motif matrix to predict the most probable amino acids of the peptide core regions, and a machine learning tool used to predict binding affinities for this system. The third and fourth methods are a previously benchmarked structural/dynamical approach using an MD/scoring and backrub/scoring combination to rank peptides bound to the MHC class II (Ochoa et al., 2019). Finally, a Molecular Mechanics-Poisson Boltzmann Surface Area (MM-PBSA) approach is used to calculate average energies per peptide based on the MD trajectories obtained in the previous strategy. In general, the predictions had an accuracy below 60% for all the methods, but combining the best scoring-matrix SM-HB (i.e., the one generated from the hydrogen bonds) with the existing methods improves the performance.

## Materials and Methods

In the following, we first explain how we build the scoring-matrices based on the structural observables. Then, we describe how to evaluate their impact on activity differences caused by single-point mutations on the peptide binders. This is followed by a description of the additional methods used for comparison and their combination with the developed structural scoring-matrices.

### Structural Scoring-Matrices From Simulations

To evaluate the impact of structural interactions, we created a set of scoring matrices based on hydrogen bonds (SM-HB) and contacts (SM-C) generated between the peptide core region and residues of the MHC class II binding site. For that purpose, we first optimized the conformational sampling of MHC class II structures using the Rosetta backrub method (Davis et al., 2006) in comparison to MD simulations. Then, we modelled a large dataset of known peptide binders of the same MHC class II allele, and with the observables we generated the scoring matrices. A detailed explanation is presented below.

#### Conformational Sampling Optimization

Before checking the role of the structural interactions, we assessed the conformational sampling of the Monte Carlo backrub method in Rosetta in comparison to MD simulations to explore conformations of crystal structures of peptides bound to MHC class II alleles. We selected a set of 10 peptide-MHC class II crystal structures from the Protein Data Bank (PDB) (Berman et al., 2000) of the most widely studied allele, DRB1*01:01 (see Supplementary Text for details about the structure selection). We used this benchmark to compare molecular dynamics (MD) and Monte Carlo backrub simulations.

**Molecular dynamics:** Each crystal structure was subjected to MD simulations of 20 nanoseconds (ns) with previous minimization and NVT/NPT equilibration phases, using GROMACS v5.1 (Hess et al., 2008). The main MD parameters are described in the Supplementary Text. A temperature of 350 K was chosen to perform the simulations, allowing a fast exploration of the conformational space. Since we are interested only in the peptide-receptor interactions, all the protein atoms located at a distance greater than 12 Å from any peptide atom were restrained.

**Backrub Monte Carlo:** The same crystal structures were subjected to Metropolis Monte Carlo simulations using the backrub algorithm (Davis et al., 2006) available in RosettaCommons version 2016.32 (www.rosettacommons.org). A total of 50,000 Monte Carlo trials were run per complex using two kT values: 0.35 and 1.2. C_*α*_ atoms were chosen as pivots for all the protein and peptide residues. The minimum backrub segment size in atoms was 3, and the maximum segment was 64. The probabilities for sampling side chain and backbone torsions were set at the default values. The simulations were run over a single core for each complex. An optimal backrub parameter setup was selected in order to reproduce the equilibrium ensemble from MD.

#### Structural observables

Several structural observables were used to characterize the conformations from the different simulations:

**Side chain dihedrals:** The side chain dihedrals $\chi_{1}$ and $\chi_{2}$ were monitored for all the amino acids belonging to the peptide, and the distributions were compared to that obtained from MD. The Kullback-Leibler divergence metric (Hershey and Olsen, 2007) was implemented to compare the distributions.

**Main chain hydrogen bonds:** We monitored interactions made by the amino acids of the peptide core region with the receptor. Specifically, we calculated the number of potential hydrogen bonds made by the backbone atoms using the HBPLUS program (McDonald and Thornton, 1994).

**Contacts:** We also calculated the number of non-bonded contacts with a threshold of 4 Å between the atoms of the peptide and those of the receptor using Biopython modules (Cock et al., 2009).

The latter two observables were also used to calculate the structural descriptors for creating the scoring-matrices.

#### Modelling and Simulations of a Large Dataset of Peptides Bound to MHC Class II

After establishing the best backrub simulation setup, a set of peptides with available binding data for different MHC class II alleles was modelled and simulated to calculate scoring-matrices from the chosen structural descriptors.

First, we selected as a representative structure of the allele DRB1*01:01 and the crystal structure with PDB id 1T5X, which is co-crystallized with a peptide that we used as template to model peptide binders with bioactivity information available. For the peptides, we used a public dataset containing 44,541 measured affinities covering 26 MHC class II alleles (Wang et al., 2010). We selected a total of 837 15 mer peptides for the allele DRB1*01:01 after applying the filter below, with activities from 1 to 10,000 nM. The filter was the prediction of the 9 mer core region of each selected peptide using two methods. The first was based on available motifs derived from a position-specific scoring matrix published for several MHC class II alleles (Rapin et al., 2008). The sequences were analyzed over windows of nine amino acids, where each fragment was scored to obtain a ranked list of fragments with probabilities of being the core region of the peptide interacting with the MHC class II receptor. For the second method, we implemented the NetMHCIIpan-4.0 tool, which has as its main goal the prediction of affinities for peptides bound to MHC class II molecules, and also the prediction of the 9°mer core regions of the peptide sequences (Andreatta et al., 2015). A peptide was selected when both methods predicted identical core regions with the highest scores. As a final step, we aligned the predicted core region with the core from the peptide template. If, after the alignment, we needed to add more than two amino acids for either flanking region (N or C-terminal), the peptide was discarded.

We modelled the selected peptides by iterative single substitutions of the peptide template sequence. The mutations were performed with the package fixbb from Rosetta (Loffler et al., 2017), which was compared in a previous study to other available mutation protocols (Ochoa et al., 2018). The method selects the most probable rotamer from a dictionary of backbone-dependent conformations. After each mutation, the side chain atoms were relaxed with the backbone fixed. The modelling of additional amino acids in the flanking region, when required, was done with the Remodel package from RosettaCommons (Huang et al., 2011), where the new amino acid was subjected to the prediction of the rotamer with relaxation of the side chains.

**FIGURE 1:**
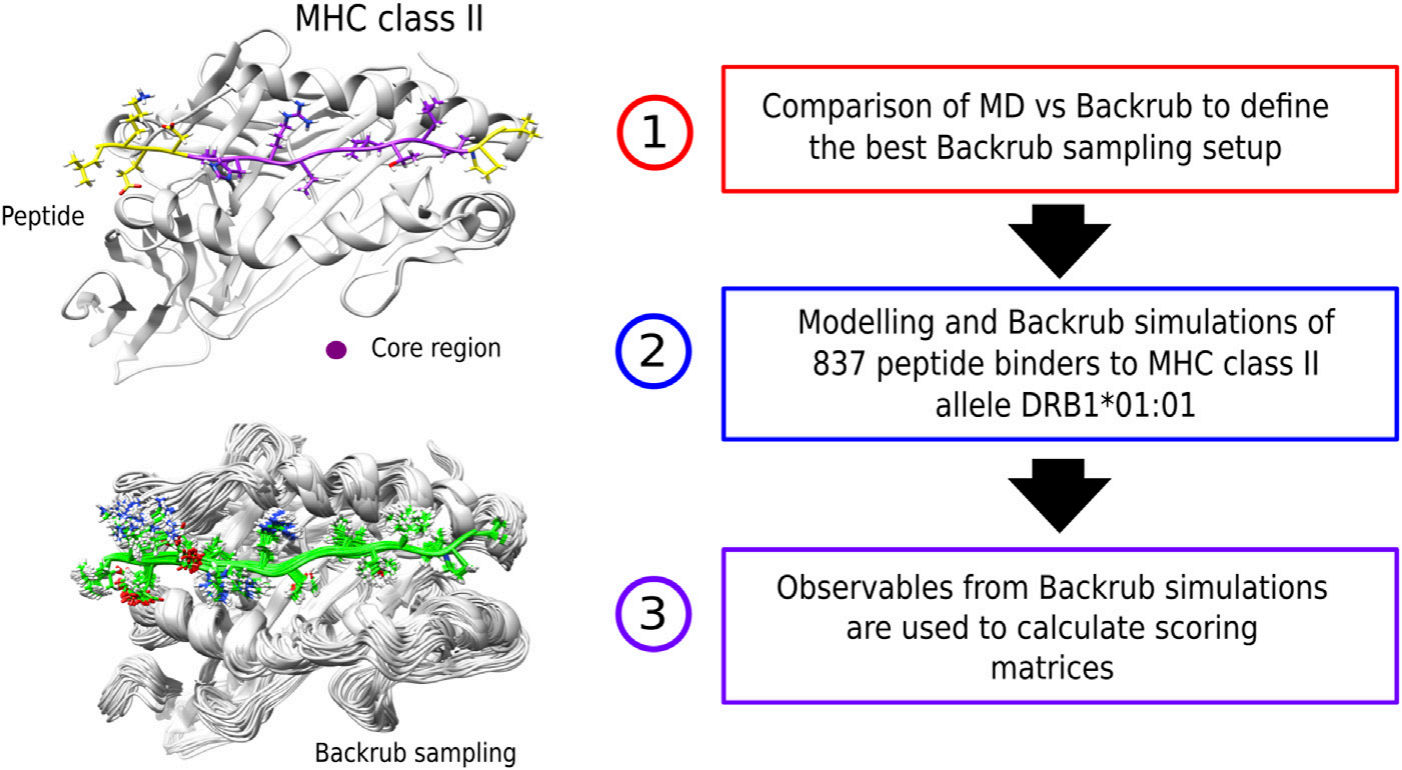
**(left)** Examples of a peptide bound to an MHC class II receptor and conformations from the Backrub Rosetta simulations. **(right)** Schematic representation of the methodological steps that involve creation of the scoring-matrices. First, an MD vs. Backrub comparison was performed to define the best Backrub setup. Then, the modelling and sampling of a set of known peptide binders was performed to obtain the observables for building the scoring-matrices.

For each peptide, the backrub simulation from Rosetta was applied with kT=1.2 (Smith and Kortemme, 2008) as found to be optimal (see the Results). Each Monte Carlo simulation had 200,000 trials. We obtained 2,000 frames per simulation, and the previously described interactions (see Methods *Structural observables*) were calculated per amino acid in all the core positions for each frame. We did the same under three other scenarios: (i) using the last 1,000, (ii) using the 1,000 frames with best energy-scores [i.e., backrub scoring function (Alford et al., 2017)], and (iii) using the single frame with the lowest energy. A summary of the modelling and sampling strategies is shown on Figure 1.

#### Definition of the Scoring-Matrices

We calculated averages of the observables per amino acid in each position of the core to define scoring-matrices of the structural descriptors. The averages covered the number of amino acids available in the dataset per position in the core region. For each position in the core region, we calculated a vector with 20 indices (one per each natural amino acid) using the average of the observable from the backrub trajectory. At the *i*th core position for amino acid type *j*, the average observable O is defined as

$$O_{ij}=\frac{1}{{\text{N}}_{\text{f}}}\underset{\alpha }{\sum }\sum_{\text{f}}o_{ij}^{\alpha \text{f}},$$ (1)

where *o* is the observable, f is the frame number, ${\text{N}}_{\text{f}}$ is the total number of frames and α indexes the simulation run (having one simulation for each binding-peptide from the dataset). In the case of using just one frame from the backrub trajectory, only the average across the simulation is calculated. If an amino acid type is not found at a given position for a certain run, the observable is taken as zero. We note that the amino acid distribution is not homogeneous but it is given by the natural-occurring frequencies found in the dataset. These intrinsic frequencies are implicitly taken into account in Eq. 1. This allows us to improve the available motifs of peptides binding to MHC class II alleles by adding weights due to the structural observables.

### The Scoring-Matrix for a Given Observable is Defined as

$$E_{ij}=-\text{ln}\frac{O_{ij}}{\sum_{j}O_{ij}}$$ (2)

which provides a scoring-energy for each amino acid (*j*) in each core position (*i*).

To visualize the frequency contribution of each amino acid on the peptide library and the scoring-matrices, logoplots were generated using the WebLogo3 server (Crooks et al., 2004).

### Assessment of Single-point Mutation Activity Predictions

The obtained scoring-matrices, SM-HB and SM-C, were compared to other methods based on their capability to predict single-point mutation activity differences. The test consisted of predicting the sign of the experimental ΔΔG for each pair of peptides differing by single-point mutations in the peptide core region. A total of 112 peptides forming 56 pairs were selected and not used to calculate the scoring-matrices from the descriptors. One requirement to select the pairs of peptides is the prediction of identical core regions with high reliability, based on the same criteria used to model the peptides (see *Modelling and simulations of a large dataset of peptides bound to MHC class II*).

### Additional Methodologies for Comparison

Five additional methods were used to compare and complement the results with the scoring-matrices. These methods are:

A sequence motif reported for allele DRB1*01:01 was used to compare the probabilities of finding an amino acid in the core region (Rapin et al., 2008). The higher the value in the matrix indicates a higher probability. The difference in probabilities was used to compare the differences in affinity.

The tool NetMHCIIpan was used to predict a numerical affinity per peptide. The sign of the predicted difference is compared to the sign of the experimental values for assessing the performance.

A hybrid MD/scoring approach was also used to predict the sign of the activity difference using structural models of the peptides based on a previously published protocol (Ochoa et al., 2019). In summary, each peptide was subjected to MD simulations of 10 ns using the same MD setup as explained previously. Each frame of the last half of the trajectory was scored using six different scoring functions: Haddock (Dominguez et al., 2003), Vina (Trott and Olson, 2009), a combination of DFIRE and GOAP (DFIRE-GOAP) (Yang and Zhou, 2008; Zhou and Skolnick, 2011), Pisa (Krissinel and Henrick, 2007), FireDock (Andrusier et al., 2007), and the BMF-BLUUES scoring combination (Berrera et al., 2003; Fogolari et al., 2012). If three or more scoring functions predicted the sign of the score differences equal to the sign of the experimental activity differences, it was counted as a match to assess the performance.

A hybrid backrub/scoring approach as explained in the previous strategy, using 50,000 Monte Carlo trials per run with a *kT* of 1.2. The backrub trajectory was scored using the same scoring functions and consensus criterion to match the sign of the activity difference.

Finally, as the most exhaustive approach, we calculated average energies per peptide complex using the MM-PBSA methodology. For that purpose we used the g_mmpbsa plugin (Kumari et al., 2014) to calculate the solvated and non-solvated terms using as input the MD trajectories of 10 ns calculated in the third strategy.

#### Combination With the Structural Scoring-Matrices

To improve the performances, we combined the previous approaches with the scoring-matrices results. Specifically, we evaluated if using the scoring-matrices together with other methods can increase the number of predictions after checking by pairs if either of the two methods predicts correctly the sign of the mutation activity difference. This analysis works as a conditional “or” to evaluate how many cases can be covered using more than one method, and subsequently observe how many predictions match the experimental data.

## Results

To evaluate the impact of interactions in affinity changes caused by single-point mutations in MHC class II peptide binders, a set of scoring-matrices was calculated to assign probabilities for each type of amino acid in each position of the peptide core region. The matrices are created using the main chain hydrogen bonds (SM-HB), and the non-bonded contacts (SM-C) obtained from trajectories of peptides in complex with the MHC class II allele. To optimize the sampling, we first compared the Backrub approach to the results from MD simulations, in order to guarantee enough conformational exploration with computationally efficiency.

### Optimization of the Structural Scoring-Matrices

#### Backrub Simulation Optimization

**FIGURE 2:**
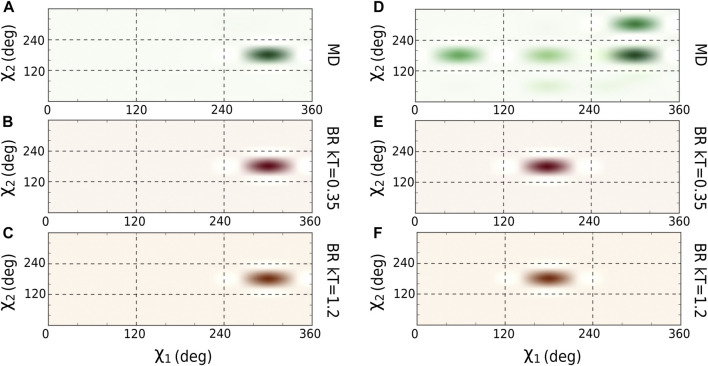
Comparison of $\chi_{1}$ and $\chi_{2}$ distributions for amino acid Leu9 from the peptide bound to MHC class II (PKYVKQNTLKLAT PDB id: 1fyt). **(A)** Last 10 ns of MD, **(B)** Backrub using kT = 0.35, and **(C)** Backrub using kT = 1.2. The same analysis was done for amino acid Arg15 of another peptide (AAYSDQATPLLLSPR PDB id 1t5x). **(D)** Last 10 ns of MD, **(E)** Backrub using kT = 0.35, and **(F)** Backrub using kT = 1.2.

We optimized the kT parameter used in backrub Rosetta simulations in comparison to finite temperature MD simulations. We used as benchmark a set of 10 peptide-MHC class II structures of allele DRB1*01:01, available in the PDB (see Supplementary Text, Supplementary Table S1; Supplementary Figure S2). After subjecting the crystal complexes to both sampling methods, the trajectories were analyzed based on several structural observables for different kT. First, we calculated the distributions of the $\chi_{1}$ and $\chi_{2}$ for each amino acid of the peptide. Examples comparing the results of MD to backrub sampling using kT=0.35 and kT=1.2 are shown in Figure 2 for two amino acids. Supplementary Table S1 shows how many amino acids were sampled similarly between the backrub and MD configurations using the side chain-dihedral distributions (see Supplementary Text and Supplementary Figure S3 for details and additional validations). We find that using the backrub simulations with kT=1.2 is suitable to efficiently explore the side chain dihedrals in comparison to MD.

We then calculated the average of the number contacts and the number of hydrogen bonds created by the main chain atoms (Table 2) for the amino acids located in the core region and for both sampling methodologies (using the optimal kT for backrub Rosetta). The fractional error calculated using standard deviation of the simulations is also shown in Table 2. We find that the averages for the backrub method are slightly lower than those for MD but within error estimates. Correlations of the values are shown in Supplementary Figure S4. The impact of the selected structural descriptors will be discussed in later sections.

**TABLE 1:** Percentage of amino acids per backrub configuration (kT=0.35 and kT=1.2) for each side chain dihedral that sampled the conformational space similarly to MD simulations among all the 10 MHC class II crystal structures

Side chain dihedrals	kT = 0.35 (%)	kT = 1.2 (%)
$\chi_{1}$	19.2	80.8
$\chi_{2}$	12.9	87.1

**TABLE 2:** Average and fractional error of the number of contacts and hydrogen bonds (HB) made by the main chain atoms of the peptide-core amino acids bound to MHC class II, and sampled with MD or backrub (BR) using kT=1.2. The fractional error was calculated using the standard deviation from the simulations for each peptide core position. The last row shows an average value for all the structures.

PDB	MD contacts	BR contacts	MD HB	BR HB
1fyt	147.9 ± 12.5	148.1 ± 9.8	8.7 ± 1.1	7.4 ± 1.1
1klg	112.6 ± 9.9	104.0 ± 7.8	8.5 ± 1.1	8.3 ± 1.2
1sje	130.3 ± 10.5	104.5 ± 7.3	10.5 ± 0.9	8.6 ± 1.1
1sjh	115.1 ± 10.6	107.8 ± 9.9	8.4 ± 1.1	9.9 ± 1.0
1t5x	135.4 ± 11.8	72.5 ± 10.1	7.8 ± 1.0	3.7 ± 1.0
2fse	126.1 ± 13.0	96.8 ± 8.9	8.9 ± 1.1	6.4 ± 0.8
3pgd	129.9 ± 13.5	133.3 ± 9.4	9.2 ± 1.1	8.7 ± 0.7
4aen	114.6 ± 11.3	99.0 ± 7.6	8.2 ± 1.2	7.4 ± 1.1
4i5b	134.8 ± 10.8	126.5 ± 9.6	8.9 ± 1.2	8.1 ± 1.1
4ov5	161.1 ± 13.8	133.9 ± 11.0	10.2 ± 1.2	8.5 ± 0.7
Average	130.7 ± 11.8	112.6 ± 9.2	8.9 ± 1.1	7.7 ± 1.0

#### Scoring-Matrices From Optimized Backrub Simulations

We selected a total of 837 15-mer peptides from the chosen bioactivity dataset (Nielsen et al., 2010) after filtering, as described in the Methods. The peptides were simulated with backrub using kT=1.2. The number of hydrogen bonds made by the main chain, and number of non-bonded contacts were calculated from the structure in the trajectories. These observables were averaged and used to calculate the scoring-matrices SM-HB and SM-C per amino acid in the core region according to the equations in the Methods.

**FIGURE 3:**
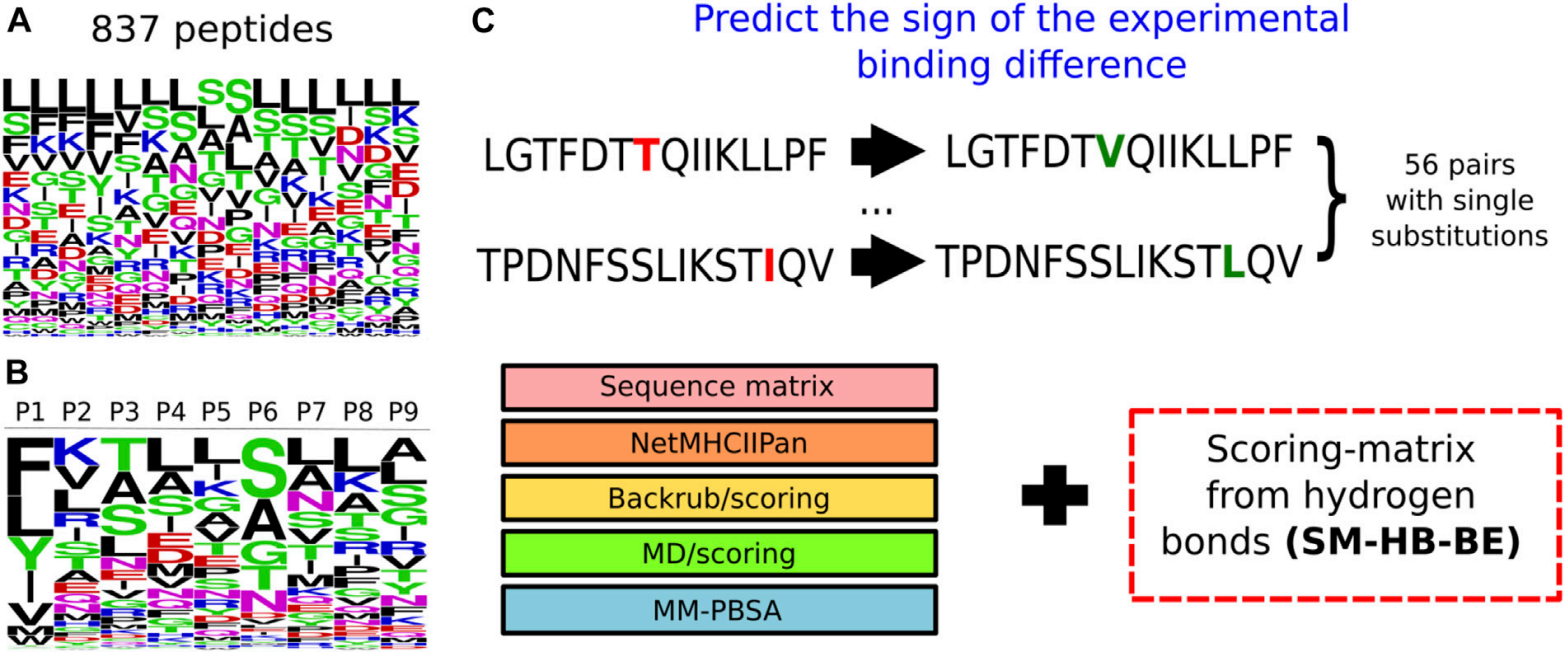
Information used to model peptides bound to the structure of the MHC class II allele DRB1*0101. **(A)** Logo representing the frequency of the amino acids within the 837 15-mer peptides that were modelled bound to the MHC class II structure. The larger the height of the letter the more relevant the amino acid is for improving binding. **(B)** Logo representing the probability of the amino acids at each position of the core region based on the number of hydrogen bonds made by the main chain. The colors represent categories of the amino acids based on physicochemical properties: blue (positive charged), red (negative charged), green (small), fucsia (asparagine) and black (aliphatic). **(C)** Prediction of the sign of the experimental binding differences, for a set of 56 peptides with single substitutions, using the scoring-matrix (SM-HB-BE) in combination with the state-of-the-art methodologies.

These scoring-matrices incorporate the frequency of the structural descriptors obtained from all the sampled peptides, as well as the amino acid distribution of the peptide library. In Figure 3, we show the frequency of the amino acid distribution in the set of 837 peptides (Figure 3A) and the motif of the peptide core region obtained from the SM-HB as observable (Figure 3B). The motif for the SM-C is available in the Supplementary Figure S5.

### Assessment of the Scoring-Matrices to Predict MHC II- Peptide Activity Differences

We first assessed if the scoring-matrices are able to predict correctly the sign of activity differences by single-point mutations on the peptide core region. The 56 pairs of peptides differing in single amino acids are reported in the Supplementary Table S2 with the corresponding experimental activities per peptide, and the difference values. This information was obtained from the bioactivity dataset (Wang et al., 2010), which follows experimental gold-standard protocols for binding measurements to MHC receptors, in comparison to other techniques (Kastritis and Bonvin, 2010). We note that this set of peptides was not included during the creation of the scoring-matrices.

The prediction results were assessed using the SM-HB and SM-C matrices with a different number and type of frames selected from the backrub trajectories. Specifically, the matrices were obtained using all the frames, the last half of the frames, half frames with best energy-scores and the single best energy frame after optimization (see Methods). A summary of the performances to predict the sign of the activity differences is shown in Table 3.

**TABLE 3:** Prediction of the sign of the experimental activity differences by single-point mutations of the peptide core amino acids using the scoring-matrix calculated based on the hydrogen bonds made by main chain atoms (SM-HB) and the number of non-bonded contacts (SM-C). The comparisons include data for the four strategies to extract information from the 2,000 backrub frames.

Strategy	Matches for SM-HB (%)	Matches for SM-C (%)
All the frames	0.553	0.501
Last half frames	0.518	0.464
Half frames with best energies	0.589	0.501
Best energy frame	0.464	0.518

We find that, in general, the observable with the highest number of correct predictions is the SM-HB, in comparison with the SM-C. In particular, for the SM-HB, the best performance was 58.9% using half of the frames with the best predicted energies based on the Rosetta scoring function (henceforth SM-HB-BE with “BE” for best energies). To complement the analysis, we calculated the scoring-matrices six times by dividing the original 837 peptide set into six independent sets. With these matrices, we calculated the mean and standard deviation of the number of matches against the experimental data (see Supplementary Table S3). In agreement with the results shown in Table 3, we found that the selected SM-HB-BE has the best performance.

### Prediction of Activity Differences for Methods That Range in Computational Costs

We compared the best structural scoring matrix (SM-HB-BE), to five previously benchmarked approaches to rank MHC class II peptide binders based on their predicted affinities, or based on the probabilities of finding certain amino acids in the peptide sequence (Figure 3C). These methods differ in the theory and, importantly, in their computational cost. In the case of the sequence-based methods, these are able to predict affinities in just a few minutes, but they largely depend on the chemical space of the training data to be successful. The structure/dynamics-based methods range from days to weeks in computational costs. The latter do not rely on training datasets but on physical, chemical and dynamical properties. To assess these diverse methodologies, we tested them to predict the sign of activity differences by single-point mutations as explained in Methods, and compared their computational cost by running them on an Intel Xeon 24-core server with NVIDIA Titan X GPU acceleration (Table 4). In addition, a bootstrapping approach with 50 replicas was ran using randomly, and with repetitions, any pair from the total 56 pairs mutated peptides, in order to obtain a standard deviation of the match for each strategy.

**TABLE 4:** Match values and bootstrapping standard deviations for the prediction of the sign of the experimental activity differences by single-point mutations of the peptide core amino acids for five state-of-the-art methodologies and the SM-HB-BE (i.e., scoring matrix from hydrogen bonds using half of the conformations with best energies). In addition, we include the computational costs, in days, for running the methods with the 56 pairs of mutated peptides. The strategies are the sequence motif matrix, the machine learning tool NetMHCIIpan, the MD/scoring and backrub/scoring approaches, and the MM-PBSA calculations (see Methods).

Complementary strategy	Matched predictions	Computational cost (days)
Sequence matrix	0.393 ± 0.067	0.05
NetMHCIIpan	0.536 ± 0.079	0.1
Backrub/scoring	0.536 ± 0.067	2
MD/scoring	0.571 ± 0.071	15
MM-PBSA	0.518 ± 0.062	15
SM-HB-BE scoring matrix	0.589 ± 0.065	0.05

We found that SM-HB-BE has a similar but slightly better match than the main state-of-the-art method (NetMHCIIpan) and the structural MD/scoring and backrub/scoring approaches, but with lower computational times. In the case of MM-PBSA, the results are similar to the backrub/scoring method, but with a computational performance that is 150 times larger than the most efficient sequence-based method (which is inconvenient for large-scale analysis). Based on the results, it is possible to use some of these structural descriptors to pre-select mutations in the core region that could improve the binding affinity requiring low computational costs. We note that the implementation of the scoring-matrices is highly efficient due to its usage as sequence-based descriptors of a particular peptide. The same happens with the sequence-based matrix and the machine learning method. In this sense, using the backrub trajectories to calculate consensus average scores is the most efficient alternative, based on time differences between a few hours to weeks taken by the backrub method and MD simulations (Table 4).

We also studied if for certain mutations their activity differences are more difficult to predict. We found that those involving arginine and charged amino acids are more challenging. In addition, amino acids changing drastically in size can misguide the predictions for the majority of the methods. A list of the cases where most of the methods fail is shown in Supplementary Table S4. Overall, these results indicate that predicting the activity differences of single-point mutations of peptides bound to MHC class II is challenging, even using extensive calculations such as MM-PBSA.

### Combining Structural Scoring-Matrices With Alternative Methodologies Improves the Affinity Difference Prediction

Because there is still room to improve the affinity-difference prediction, we combined the results of each additional method with the SM-HB-BE. The combination consists on checking if either of the two methods predicts a positive mutation, if so then the mutation has a match with the experimental data. This allow us to verify which method complements better with the selected scoring-matrix (Table 5). We also included the standard deviations of the matches by following the same bootstrapping approach explained in the previous section.

**TABLE 5:** Match values and bootstrapping standard deviations for the prediction of the sign of the experimental activity differences by single point mutations of the peptide core amino acids. The results are for the combination of the additional methodologies with the SM-HB-BE matrix. The strategies are the sequence motif matrix, the machine learning tool NetMHCIIpan, the MD/scoring and backrub/scoring approaches, and the MM-PBSA calculations (see Methods).

Complementary strategy	Matched predictions in combination with the SM-HB-BE matrix
Sequence matrix	0.607 ± 0.062
NetMHCIIpan	0.714 ± 0.064
Backrub/scoring	0.786 ± 0.051
MD/scoring	0.857 ± 0.047
MM-PBSA	0.786 ± 0.048

We found that combining SM-HB-BE with the MD/scoring approach can predict correctly 85.7% of the mutations included in the study, followed by a 78.6% using the backrub/scoring and the MM-PBSA methodologies. As seen, the best results are found after combining the scoring-matrices with structure/dynamic-based strategies, but such combination can be done with the backrub/scoring approach that is more computationally efficient (Table 4). In any case, the calculated scoring-matrices can improve information about the frequency of amino acids in core positions using motif representations, and overall the performance is higher than using the sequence-based matrices available in the literature (Rapin et al., 2008).

## Discussion

We evaluated the role of structural observables from simulations for predicting activity differences caused by single-point mutation of MHC class II peptide binders. A scoring-matrix derived from counting the number of hydrogen bonds formed by the main chain atoms using the best Rosetta energies (SM-HB-BE), can significantly improve the prediction of these differences if combined with other sequence or simulation-based methodologies.

To deal with the number of modelled peptides, we required running an efficient methodology for sampling the conformations as closely as possible to their equilibrium ensemble. After optimizing the Monte Carlo backrub parameters, we obtained similar conformations to those explored by MD simulations. We note that the MD simulation time was chosen based on previous assessments for exploring well the conformations around the mutated complex, which is around 20 ns for this system (Ochoa et al., 2019; Ochoa et al., 2020). The backrub method tends to perform a similar exploration of the formation of certain contacts and hydrogen bonds, mostly those created by the core region of the peptide. Moreover, the RMSD values between conformations from MD vs backrub are indistinguishable from those of MD vs MD. However, we note that the method is unable to reproduce completely the landscape explored by MD, which can be a limitation. This is why starting from a crystallized bound-conformation is critical for providing more reliable poses of the modelled peptides. Regarding the computational time, the backrub method can sample a similar number of frames as MD in just a few hours, in comparison to days required for MD in high-computing infrastructures (Table 4). This facilitates the analysis of a large set of peptides for this MHC class II allele, and others with structures available in public databases.

The peptide were selected based on criteria that facilitate the initial modelling of the rotamers (Ochoa et al., 2018), and the inclusion, in some cases, of additional flanking amino acids. Moreover, these peptides have available experimental binding data. Therefore, the new descriptors contain intrinsic information about the distribution of amino acids based on binding information, implying that our structural insights are complementing the known sequence-based motifs (Menconi et al., 2008; Andreatta et al., 2012). This is relevant because our protocol does not start from scratch. Instead, its main goal is to exploit the current knowledge of the system, and provide better metrics for the understanding of the MHC class II binding using simulations.

The calculated observables can be compared to the reported MHC class II promiscuity in terms of the intrinsic stability of the interactions between the peptide and the MHC class II binding groove. We found, for example, that the hydrogen bonds created by the main chain atoms are one of the most important structural observables. This claim has also been proposed in other studies, motivated by the stability of the peptide-bound conformation in spite of being completely linear, which is crucial in the molecular editing processes within the antigen presentation pathways (Painter et al., 2008; Yaneva et al., 2010; Ferrante et al., 2015). Therefore, simulating the dynamics of the complex can bring novel insights into the binding nature, and allows us to predict activity differences caused by single-point substitutions on the peptide sequence.

## Conclusion

Simulations provide structural insights for creating simple scoring-matrices that complement available methods to better predict the effect of single-mutations on the binding of peptides to MHC class II molecules. Integrating sequence, structural and dynamical information is useful to progress in the immunoinformatics field, not only for MHC class II structures, but also for other key components within the immune response pathways.

Moreover, the methodology can contribute to the identification of epitopes for certain alleles using structural and dynamical information. In fact, the method can be expanded to calculate descriptors for other peptide-binding complexes, to design of novel epitopes by single-point substitutions, and to understand the impact of antigen mutations in the immune system, for example, using structural interactions with the *T*-cell receptors, having direct consequence in vaccine design (Purcell et al., 2007). The descriptors can also help to, possibly, discriminate at a reasonable level between good binders and non-binders. However, a better discrimination requires combining multiple methods, or implement more exhaustive approaches to capture the chemical contributions of the peptide residues through more explicit free energy calculations (Wieczorek et al., 2016; Huang et al., 2017).

## Data Availability

The datasets presented in this study can be found in online repositories. The names of the repository/repositories and accession number(s) can be found below: https://github.com/rochoa85/MHC_class_II_matrices, Github.
