# Supplementary material for: Impact of Structural Observables From Simulations to Predict the Effect of Single-Point Mutations in MHC Class II Peptide Binders
Source: Front Mol Biosci. 2021 Mar 30;8:636562. doi: 10.3389/fmolb.2021.636562 (PMC8253603; doi:10.3389/fmolb.2021.636562)
Supplement: Supplementary file 1 [file datasheet1.pdf]

frontiers

# *Supplementary Material*

## 1 SUPPLEMENTARY TEXT

### 1.1 MD parameters

For the simulations, the Amber99SB-ILDN protein force-field (Lindorff-Larsen et al., 2010), a TIP3P water model (Jorgensen et al., 1983), a modified Berendsen thermostat (Bussi et al., 2007), and a Parrinello-Rahman barostat (Parrinello and Rahman, 1980) were used during the equilibration and production phases. The complex was solvated in a cubic box of water with periodic boundaries at a distance of at least 8Å from any atom of the protein. Counterions of $Na^+$ and $Cl^-$ were included in the solvent to make the box neutral. The electrostatic interactions were calculated using the Particle Mesh Ewald (PME) method, with 1.0 nm short-range electrostatic and van der Waals cutoffs (Di Pierro et al., 2015). The equations of motion were solved with the leap-frog integrator (Janežič and Merzel, 1995), using a timestep of 2 femtoseconds (fs).

### 1.2 Peptide-MHC class II crystal structures for backrub optimization

We selected 10 peptide-MHC class II crystal structures for optimizing the backrub Rosetta simulations. The selection was based on previous annotated data of the bound peptides and their core regions (Andreatta et al., 2015). Information about the publication year, the crystal resolution, experimental B-factors and occupancy of the atoms were reviewed to verify the quality of the structures. The information for each complex about crystal resolution, publication date, peptide sequence and reported core region is available in Supplementary Table S1. In the case of missing residues for the peptide or protein-binding interface, backbone or side chain atoms were modelled using the Rosetta Remodel package (Huang et al., 2011). After the required edits, all the structures were relaxed with backbone atoms fixed. A structural alignment was made to compare the conformations of the bound peptides (Supplementary Figures S2A and B).

### 1.3 Backrub simulation optimization

To quantify which $kT$ is more appropriate for the system, the dihedral angle distributions for each amino acid position were compared using the Kullback-Leibler (KL) divergence metric. Specifically, the histograms for each amino acid dihedral were split into 3 bins, one per rotamer group (Wolfe, 1972), and normalized. The divergence values were compared, and those closest to zero were used to determine which backrub configuration was best to explore the MD configurations. These results are presented in Table **??**. We find that the exploration can be increased by elevating the $kT$.

As an additional check, we calculated the peptide $C_\alpha$-RMSD between the conformations from the backrub with $kT$=1.2 and the MD simulations. We compared the distribution to the conformations of MD with itself. We find that the distributions significantly overlap, showing that the probability of picking a conformation from the backrub method is similar to choosing one from the MD simulation (see Supplementary Figure S3).

## 2 SUPPLEMENTARY FIGURES AND TABLES

### 2.1 Figures

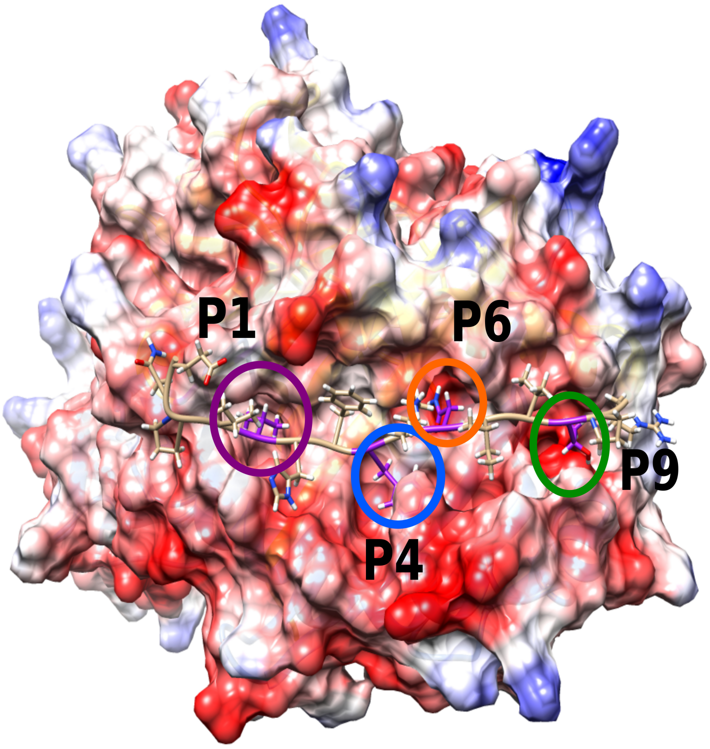


**Figure S1.** MHC class II bound to a peptide. The PDB structure id is 1BX2. The interface is characterized by the key pockets P1 (purple circle), P4 (blue circle), P6 (orange circle) and P9 (green circle). The amino acids from the peptide in contact (<3 Å) with the receptor pockets are colored in violet.

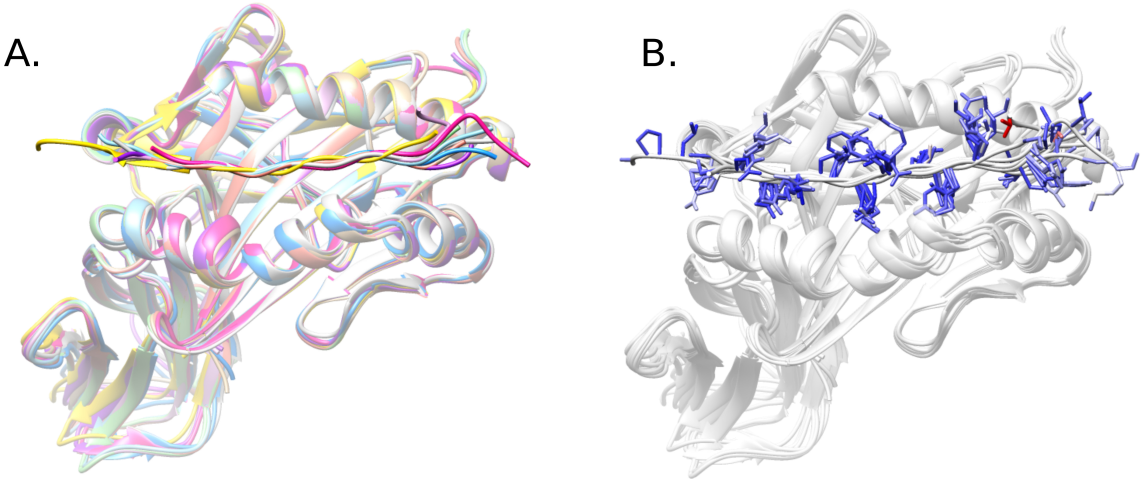


**Figure S2.** (A) Structural alignment of the crystallized MHC class II structures of allele DRB1*01:01. (B) B-factors colored, where red is flexible and blue is rigid.

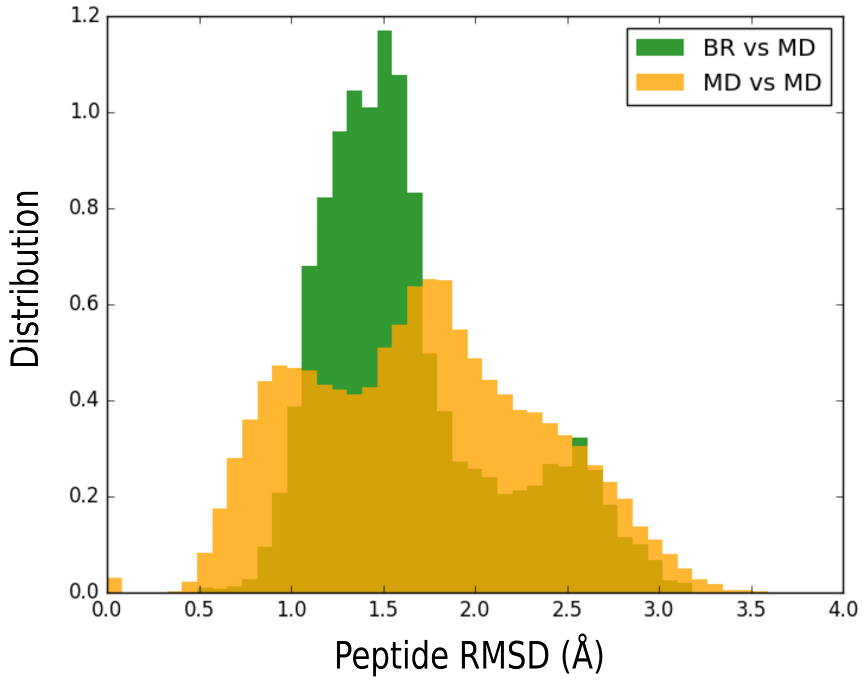


**Figure S3.** Distributions of the peptide Cα-RMSD between conformations obtained from the backrub trajectory using kT=1.2 with MD (green), and MD with itself (orange). Both distributions overlap in a similar range of RMSD values.

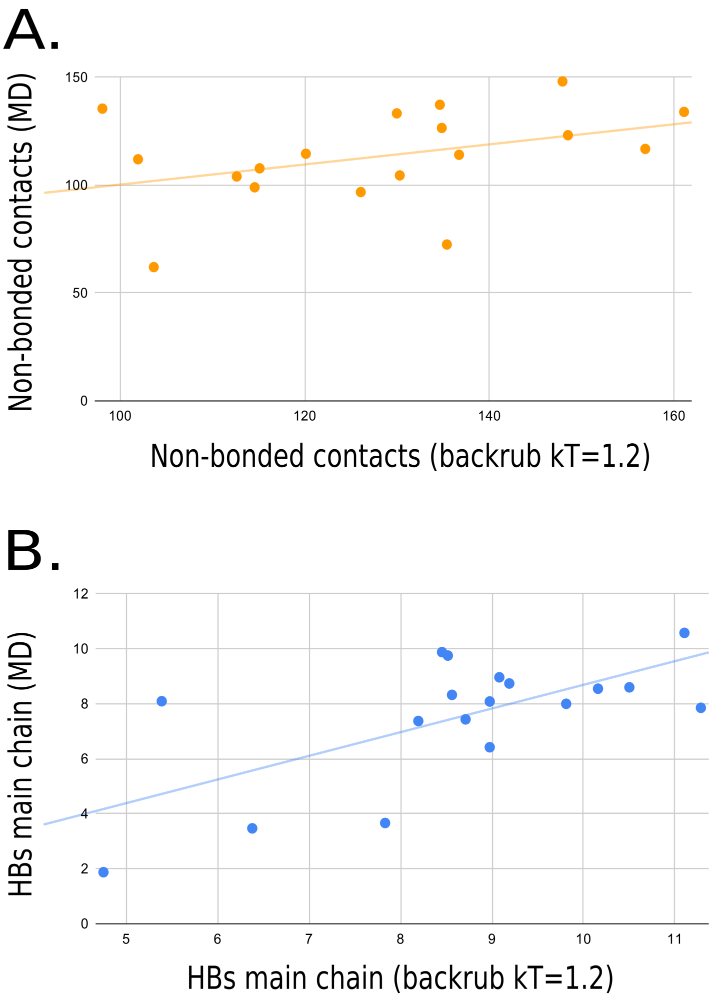


**Figure S4.** Correlations of the sum of the number of contacts (A) and hydrogen bonds (HB) by the main chain atoms (B) of the peptide core regions bound to MHC class II structures sampled with MD and backrub using kT=1.2.

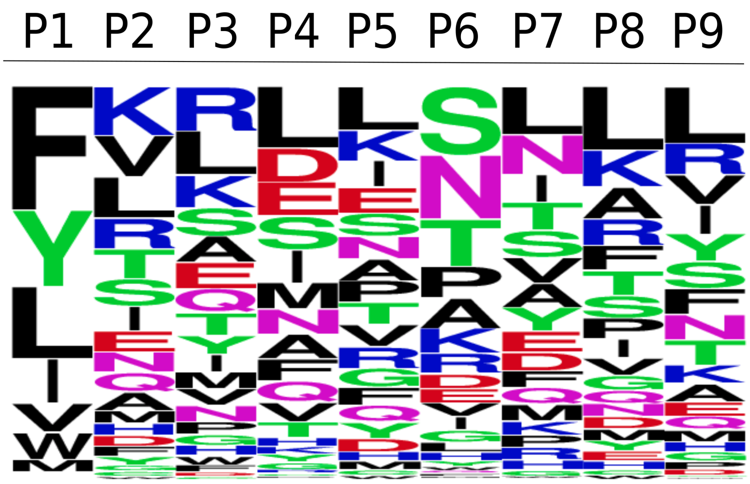


**Figure S5.** Logo representing the probability of the amino acids at each position of the core region based on the number of non-bonded contacts. The larger the height of the letter the more relevant the amino acids is for improving the binding, The colors represent categories of the amino acids based on physico-chemical properties: purple (positive-charged), red (negative-charged), green (small), fucsia (asparagine) and black (aliphatic).

**Table S1.** List of MHC class II structures of the allele DRB1*01:01 bound to peptides that were used to compare backrub to MD sampling. Information about the crystal resolution, publication year and sequences of the full and core regions are reported.

| PDB | Resol | Year | Full peptide | Core |
|---|---|---|---|---|
| 1fyt | 2.6 | 2000 | PKYVKQNTLKLAT | YVKQNTLKL |
| 1klg | 2.4 | 2002 | GELIGTLNAAKVPAD | IGTLNAAKV |
| 1sje | 2.45 | 2004 | PEVIPMFSALSEGATP | VIPMFSALS |
| 1sjh | 2.25 | 2004 | PEVIPMFSALSEG | VIPMFSALS |
| 1t5x | 2.5 | 2004 | AAYSDQATPLLLSPR | YSDQATPLL |
| 2fse | 3.1 | 2006 | AGFKGEQGPKGEPG | FKGEQGPKG |
| 3pgd | 2.72 | 2010 | KMRMATPLLMQALPM | MRMATPLLM |
| 4aen | 2.2 | 2012 | MKMRMATPLLMQALPM | MRMATPLLM |
| 4i5b | 2.12 | 2013 | VVKQNCLKLATK | VVKQNCLKL |
| 4ov5 | 2.19 | 2014 | GSDARFLRGYHLYA | ARFLRGYHL |

## 2.2 Tables

**Table S2.** Pairs of peptides differing by single-point mutations with available binding activity data against the MHC class II allele DRB1*01:01. The activity values and differences are given in nanomolar (nM) units.

| Peptide 1 | Activity 1 | Peptide 2 | Activity 2 | Activity difference |
|---|---|---|---|---|
| LGTFDTTQIIKLLPF | 642.533 | LGTFDTVQIIKLLPF | 137.049 | 505.484 |
| TPDNFSSLIKSTIQV | 70.020 | TPDNFSSLIKSTLQV | 17.859 | 52.161 |
| LGNFSWFPHKDMMPS | 17.492 | LGNFSWFPHKEMMPS | 7758.346 | -7740.854 |
| GWPYIGSRSQIIGRS | 1.925 | GWPYIGSRSQILGRS | 1.252 | 0.673 |
| KVVNKNIERPMFRND | 33.062 | KVVNKNVERPMFRND | 14540.722 | -14507.66 |
| LDECLHLLRTDSVFK | 156.081 | LDECLHLLRTDSIFK | 38.408 | 117.673 |
| LTKFVSAALHNVKCK | 6.581 | LTKFVAAALHNVKCK | 2.372 | 4.209 |
| DDILTLSREAVESCP | 16643.509 | DDILALSREAVESCP | 12282.285 | 4361.224 |
| TLWLDIEGPPTDPVE | 73.959 | TLWLDIEGPATDPVE | 39.417 | 34.542 |
| TLIFKGEKKLNSLDP | 16835.579 | TLIFKGEKKLNNLDP | 5094.659 | 11740.92 |
| KFFSENDWFSCMKMI | 38705.070 | KFFSEGDWFSCMKMI | 459.683 | 38245.387 |
| LLYKLCLSGEGWPYI | 14.100 | LLYKLCLSGDGWPYI | 3.544 | 10.556 |
| FEKFFEPKSQFGFFV | 2175.366 | FEKFFEPKSEFGFFV | 3.007 | 2172.359 |
| SKLKLLKGSETTVTE | 4.573 | SKLKLLRGSETTVTE | 34.405 | -29.832 |
| EEFIRLLKNRKKSKV | 3.207 | EEFIRLLRNRKKSKV | 95.482 | -92.275 |
| SIDFNQVSQVQRALR | 116.132 | SIDFNQVAQVQRALR | 8.918 | 107.214 |
| GEKKLNNLDPMTNSG | 254.006 | GEKKLNSLDPMTNSG | 774.645 | -520.639 |
| QGILHNTSDLYGLIT | 131.671 | QGILHNSSDLYGLIT | 161.957 | -30.286 |
| NSHHYISMGKSGLEL | 23.886 | NSHHYISMGTSGLEL | 29.234 | -5.348 |
| SVLLVVALFAVFLGS | 5072.752 | SVLLVVVLFAVFLGS | 24383.572 | -19310.82 |
| YFESFVREFVATART | 1.454 | YFESFVREFVATTRT | 550.264 | -548.81 |
| RTLILLMLTNPTKRN | 1.000 | RTLLLLMLTNPTKRN | 19.658 | -18.658 |
| LECFVRSTPASFEKK | 6.069 | LECFVRSSPASFEKK | 52.124 | -46.055 |
| STWYGKPTGAGPKDN | 22652.512 | STWYGKPTAAGPKDN | 8826.363 | 13826.149 |
| DPFLLNTNTDVKDWL | 39.663 | DPFLLNTHTDVKDWL | 4.743 | 34.92 |
| NSVIQALTSLGLLYT | 1.000 | NSVVQALTSLGLLYT | 1.000 | 0.001 |
| VREFVATTRTLGNFS | 444.687 | VREFVATARTLGNFS | 5.407 | 439.28 |
| GLLYTVKYPNLNDLD | 1544.854 | GLLYTVKYPNLSDLD | 302.804 | 1242.05 |
| NISYMCHFITKETPD | 570.646 | NISYLCHFITKETPD | 323.492 | 247.154 |
| IPLSFLPDWFAFKDC | 4.211 | IPLSLLPDWFAFKDC | 485.757 | -481.546 |
| SLRRLSSVCLALTNS | 10.282 | SLRKLSSVCLALTNS | 3.079 | 7.203 |
| RSILLIPLSFLPDWF | 10.395 | RSILLIPLSLLPDWF | 127.733 | -117.338 |
| FGSMPALTIACMTVQ | 1.000 | FGSVPALTIACMTVQ | 17.578 | -16.578 |
| EVLVLLEFHSHLSGL | 1084.960 | EVLVLLEFQSHLSGL | 446.304 | 638.656 |
| NKFVSPKSVSGTFVA | 2.428 | NKFVSPKSVIGTFVA | 1.000 | 1.428 |
| EAVKKLDPTNTLWLD | 13.339 | EAVGKLDPTNTLWLD | 22.365 | -9.026 |
| LETFVRVNPEEFEKK | 21.232 | LETFVRVNPDEFEKK | 300.199 | -278.967 |
| EVFCQVIKLDSEEYH | 8112.694 | EVFCQTIKLDSEEYH | 2390.000 | 5722.694 |
| LEFHSHLSGLLNKFI | 6.967 | LEFQSHLSGLLNKFI | 148.548 | -141.581 |
| CGIFALVSFLLLAGR | 10.401 | CGIFALISFLLLAGR | 5938.259 | -5927.858 |
| SDFYGLLSERFINYC | 68.462 | SDFYGLISERFINYC | 1.000 | 67.462 |
| TDDNEEPIAAYHFDL | 4867.638 | TDDNEEPIAPYHFDL | 12260.206 | -7392.568 |
| WFPHKDMMPSEDGAE | 12919.732 | WFPHKEMMPSEDGAE | 26700.961 | -13781.229 |
| IDRRMLDECLHLLRT | 22991.483 | IDRRLLDECLHLLRT | 89.505 | 22901.978 |
| KLRDLNKEVDRLMSM | 543.702 | KLRDLNKEVDNLMSM | 234.378 | 309.324 |
| RKQKYKLSHSDYEYK | 1172.631 | RKQKYKLRHSDYEYK | 203.433 | 969.198 |
| RYNRGRRANDKGDGE | 2738.959 | RYRRGRRANDKGDGE | 12363.216 | -9624.257 |
| SSMVEAMVSRARIDA | 879.191 | SSMMEAMVSRARIDA | 337.045 | 542.146 |
| NLNDLEKLKDKHPVL | 6064.416 | NLNDLERLKDKHPVL | 19221.246 | -13156.83 |
| QGEPGRVIRGKKGAG | 251.900 | QGEPGAVIRGKKGAG | 327.081 | -75.181 |
| SEDLLKAVLGAKRKL | 197.395 | SEDLLKAVLGAKKKL | 10.548 | 186.847 |
| TSLMDKLREDLITPA | 23866.960 | TSLMDKLKEDLITPA | 347.170 | 23519.79 |
| QSKLSRNFTKGVKKI | 327.687 | QSKLSKNFTKGVKKI | 289.037 | 38.65 |
| LSDLVDSDPEEVLVL | 929.579 | LSDLVVSDPEEVLVL | 33377.867 | -32448.288 |
| EITGIMKDLDEPGHL | 9513.367 | EITGIMKDFDEPGHL | 8265.463 | 1247.904 |
| LHRFRIGEHLLGSEI | 7.892 | LHRFRTGEHLLGSEI | 2591.844 | -2583.952 |

**Table S3.** Prediction of the sign of the experimental activity differences by single-point mutations of the peptide core amino acids using the SM-HB matrix. The comparisons include the average and standard deviations of the matches after splitting the peptide set into six independent groups. The calculation was made for the four strategies to extract information from the 2000 backrub frames.

| Strategy | Matches for SM-HB (%) |
|---|---|
| All the frames | $0.517 \pm 0.052$ |
| Last half frames | $0.461 \pm 0.046$ |
| Half frames with best energies | $0.548 \pm 0.061$ |
| Best energy frame | $0.441 \pm 0.058$ |

**Table S4.** Single-point mutations commonly failed by the computational methods. The specific amino acids (AA) by pairs and the number of methods that fail.

| AA Peptide 1 | AA Peptide 2 | Number of methods failing |
|---|---|---|
| R | K | 5 |
| K | R | 5 |
| Q | E | 4 |
| N | R | 4 |
| H | Q | 4 |
| D | E | 3 |
| N | S | 3 |
| N | H | 3 |
| K | T | 3 |
| S | R | 3 |
| S | N | 3 |

## REFERENCES

Lindorff-Larsen K, Piana S, Palmo K, Maragakis P, Klepeis JL, Dror RO, et al. Improved side-chain torsion potentials for the Amber ff99SB protein force field. *Proteins: Structure Function and Bioinformatics* **78** (2010) 1950–1958. doi:10.1002/prot.22711.

Jorgensen WL, Chandrasekhar J, Madura JD, Impey RW, Klein ML. Comparison of simple potential functions for simulating liquid water. *Journal of Chemical Physics* **79** (1983) 926–935. doi:10.1063/1.445869.

Bussi G, Donadio D, Parrinello M. Canonical sampling through velocity rescaling. *Journal of Chemical Physics* **126** (2007) 014101.

Parrinello M, Rahman A. Crystal structure and pair potentials: A molecular dynamics study. *Physical Review Letters* **45** (1980) 1196–1199. doi:10.1103/PhysRevLett.45.1196.

Di Pierro M, Elber R, Leimkuhler B. A stochastic algorithm for the isobaric-isothermal ensemble with Ewald summations for all long range forces. *Journal of Chemical Theory and Computation* **11** (2015) 5624–5637. doi:10.1021/acs.jctc.5b00648.

Janežič D, Merzel F. An efficient symplectic integration algorithm for molecular dynamics simulations. *Journal of Chemical Information and Computer Sciences* **35** (1995) 321–326. doi:10.1021/ci00024a022.

Andreatta M, Karosiene E, Rasmussen M, Stryhn A, Buus S, Nielsen M. Accurate pan-specific prediction of peptide-MHC class II binding affinity with improved binding core identification. *Immunogenetics* **67** (2015) 641–650. doi:10.1007/s00251-015-0873-y.

Huang PS, Ban YEA, Richter F, Andre I, Vernon R, Schief WR, et al. RosettaRemodel: A generalized framework for flexible backbone protein design. *PLoS ONE* **6** (2011) e24109. doi:10.1371/journal.pone.0024109.

Wolfe S. Gauche effect. stereochemical consequences of adjacent electron pairs and polar bonds. *Accounts of Chemical Research* **5** (1972) 102–111.
